# Hyperthyroid hypokalemic periodic paralysis

**DOI:** 10.12669/pjms.324.11006

**Published:** 2016

**Authors:** N.S. Neki

**Keywords:** Hyperthyroid periodic paralysis, Hypokalemia

## Abstract

Hyperthyroid periodic paralysis (HPP) is a rare life threatening complication of hyperthyroidism commonly occurring in young Asian males but sporadically found in other races. It is characterised by hypokalemia and acute onset paraparesis with prevalence of one in one hundred thousand (1 in 100000). The symptoms resolve promptly with potassium supplementation. Nonselective beta blockers like propranol can also be used to ameliorate and prevent subsequent paralytic attack. We report a case of 22 year old male presenting with hyperthyroid periodic paralysis (HPP) having very low serum potassium level.

## INTRODUCTION

Hyperthyroid periodic paralysis (HPP) is a rare but potentially lethal manifestation of hyperthyroidism, which mainly affect young Asian males in the age group of 20-40 years although hyperthyroidism is more commonly in females.^1^ It is characterised by acute paralytic attacks and hypokalemia in association with hyperthyroidism.^2^ HPP resolves when the patient becomes euthyroid so that the definitive treatment is either radioactive iodine or thyroidectomy. Patients usually present early hours in the morning or after rest following heavy uncustomed exercise or a high carbohydrate meal. The symptoms can last hours to days and are usually transient. Symptoms resolve promptly with correction of hypokalemia.

## CASE REPORT

A 22 year old patient, non alcoholic, non diabetic, vegetarian presented with complaints of sudden onset symmetrical weakness of lower limb muscles predominantly proximal group. He woke up in the morning to find that he was unable to move, walk and stand up. There was no respiratory distress. The patient reported palpitation, heat intolerance, excessive sweating, irritability, tremors of both hands, weight loss. On examination he was anxious, BP 130/80 mmHg, Pulse rate 90/min, respiratory rate 16/minutes. neck examination revealed diffuse thyromegaly with a bruit on auscultation. There was no opthalmopathy. Deep tendon reflexes were diminished while planter response was flexor bilaterally. Power was normal in both upper limbs but 2/5 in both lower limbs with hypotonia. There was no sensory deficit.

Cranial nerves revealed no abnormality. Heart and lung examination was non contributory. Laboratory findings including blood sugar, renal profile, liver function tests, creatinine phosphokinase, sodium, calcium and magnesium were within normal limits. Serum potassium was 2.5 meq/L, X-ray chest was normal. ECG shows sinus tachycardia. Thyroid function tests showed T3 250 ng/dl (normal 80-160), T4 20 ng/dl (normal 5-12) and TSH 2.4 mIU/L (normal 0.5-4.5). An iodide 123 thyroid uptake nuclear medicine scan showed a 2 hour thyroid uptake of 20% (normal less than 8%) and a 24 hour uptake of 50% (normal less than 33%) consistent with hyperthyroidism. He was managed with methimazole and propranolol, and IV potassium chloride. Patient responded well. No further episodes of hypokalemia or paralysis were noted.

## DISCUSSION

HPP usually affects young Asian males in their 3^rd^ decade of life.^2^ Some authors have reported HPP in a young boy of 14 years.^3^ Typical HPP attack is characterised by transient episode of muscular weakness usually involving lower limbs.^4^ The muscular weakness may range from mild weakness to total flaccid paralysis. Sensory functions, bowel and bladder were not affected. Precipitating factors of HPP include high carbohydrate diet, high salt intake, trauma, surgery, rest after sternous unaccustomed exercise, cold exposure, alcohol, emotional stress and drug like diuretics, estrogen, laxatives, steroids, amphotericin B etc. Attacks may have correlation with seasonal variation that frequent attacks occur in summer months, which may be due to increased outdoor activity and consumption of sweet drinks in the summer. Deep tendon reflexes are markedly diminished with hypotonia.^5^ Rarely patient can develop quadriparesis which needs to be differentiated from Guillian-Barre syndrome, transverse myelitis and spinal cord compression. Recurrent episodes of weakness in between the attacks may be experienced by some patients, otherwise patient usually has complete recovery.^6^ Hypothalamus is the hallmark of HPP. It occurs due to shift of potassium into the cells from extracellular space. The rapid influx of potassium is due to increased activity of sodium potassium adenosine triphosphatase pump (Na^+^ K^+^ ATPase).^7^ Increased Na^+^ K^+^ ATPase activity is augmented by insulin excess, increased adrenergic response and high circulating levels of thyroid hormones in patients of hyperthyroidism. This leads to hypokalemia and subsequent periodic paralysis.^8^ Androgens also enhance Na^+^ K^+^ ATPase pump activity.^9^ Treatment of HPP includes immediate potassium replacement therapy either orally or IV depending upon the severity of hypokalemia. But it cannot prevent acute paralysis if given between attacks.^7^,^10^ The main treatment of HPP involves control of hyperthyroidism using antithyroid drugs, radioiodine ablation or thyroidectomy^11^. Precipitating factors must be avoided. Acetazolamide has no role since it may worsen HPP.

## CONCLUSION

HPP is potentially lethal complication of hyperthyroidism occurring in Asian men in the age group of 20 - 40 years. Early diagnosis and treatment will prevent serious cardiac complications. At the time of acute attacks, treatment should be started with low dose of potassium supplements. Serial monitoring of potassium levels is recommended in order to prevent rebound hyperkalemia. All efforts should be aimed at achievement of euthyroid status as early as possible. The definitive treatment is radioactive iodine or thyroidectomy.
